# Roadway traffic crash prediction using a state-space model based support vector regression approach

**DOI:** 10.1371/journal.pone.0214866

**Published:** 2019-04-05

**Authors:** Chunjiao Dong, Kun Xie, Xubin Sun, Miaomiao Lyu, Hao Yue

**Affiliations:** 1 Key Laboratory of Transport Industry of Big Data Application Technologies for Comprehensive Transport, Ministry of Transport, Beijing Jiaotong University, Shangyuancun, Haidian District, Beijing, China; 2 National Demonstration Center for Experimental Traffic and Transportation Education, School of Traffic and Transportation, Beijing Jiaotong University, Shangyuancun, Haidian District, Beijing, China; 3 School of Electronic and Information Engineering, Beijing Jiaotong University, Shangyuancun, Haidian District, Beijing, China; 4 School of Transportation and Logistics, Southwest Jiaotong University, Jinniu District Chengdu, China; Tsinghua University, CHINA

## Abstract

Conventional traffic crash analyzing methods focus on identifying the relationship between traffic crash outcomes and impact risk factors and explaining the effects of risk factors, which ignore the changes of roadway systems and can lead to inaccurate results in traffic crash predictions. To address this issue, an innovative two-step method is proposed and a support vector regression (SVR) model is formulated into state-space model (SSM) framework for traffic crash prediction. The SSM was developed in the first step to identify the dynamic evolution process of the roadway systems that are caused by the changes of traffic flow and predict the changes of impact factors in roadway systems. Using the predicted impact factors, the SVR model was incorporated in the second step to perform the traffic crash prediction. A five-year dataset that obtained from 1152 roadway segments in Tennessee was employed to validate the model effectiveness. The proposed models result in an average prediction MAPE of 7.59%, a MAE of 0.11, and a RMSD of 0.32. For the performance comparison, a SVR model and a multivariate negative binomial (MVNB) model were developed to do the same task. The results show that the proposed model has superior performances in terms of prediction accuracy compared to the SVR and MVNB models. Compared to the SVR and MVNB models, the benefit of incorporating a state-space model to identify the changes of roadway systems is significant evident in the proposed models for all crash types, and the prediction accuracy that measured by MAPE can be improved by 4.360% and 6.445% on average, respectively. Apart from accuracy improvement, the proposed models are more robust and the predictions can retain a smoother pattern. Furthermore, the results show that the proposed model has a more precise and synchronized response behavior to the high variations of the observed data, especially for the phenomenon of extra zeros.

## 1. Introduction

Traffic crashes result in countless fatalities, injuries, many dollars expenses in medical and property lost. To reduce the number of traffic crashes, many models and approaches have been developed to investigate the relationship between the impact factors and traffic crash outcomes and intend to provide effective traffic safety countermeasures. Lord and Mannering [1] provided a comprehensive review about the methodologies that previously applied to traffic crash analyses along with their strengths and weaknesses. Though these studies provide valuable insights to enhance the traffic safety, it is important to note that crash estimation and crash prediction require different methodologies and approaches. With regards to the crash estimation, the relationships between crash counts and impact factors, such as geometric design features, traffic factors, and environmental characteristics have been analyzed in numerous previous studies [2–8].

Varieties of statistical models that are based on Poisson distribution as well as some extensions of the Poisson models, including negative binomial (NB) and Poisson-lognormal models have been proposed for traffic crash estimations over the years. These models focused on a thorough understanding of the factors that affect the occurrences of traffic crashes. To better estimate the likelihood of crashes and provide guidance for policies and countermeasures that aim at reducing the number of crashes, issues such as the effects of unobserved heterogeneities, spatial and temporal correlations, the possibility of roadway segments shifting among multiple crash states have been addressed in the evolution of methodological alternatives in the field of traffic crash analyses. To account for the unobserved heterogeneity from one roadway entity to another, the random-parameter feature has been incorporated in these generalized Poisson models and the estimated parameters are allowed to vary across each individual observation in the dataset [9]. To address the correlation issues in crash data, the bivariate/multivariate models, such as multivariate Poisson model [10], the bivariate NB model [11], the multivariate Poisson-lognormal model [12–14], the multivariate zero-inflated Poisson model [15], and the multivariate random-parameters zero-inflated NB regression model [16] have been applied in highway-safety research to jointly model more than one crash type simultaneously, since the counts of specific crash types are not independent. Zeng and Huang [17] and Zeng et al. [18, 19] addressed the spatial and temporal correlation issues in the modelling process for traffic crash analyses.

The estimated parameters of the explanatory variables do not directly show the magnitude of the effects on the expected frequency for all levels and injury severities. With the estimated parameters and an average value of individual impact factor, marginal effects can be estimated to measure a unit increase in the variable resulting in an average increase (decrease) in the number of crashes. The calculation formulas and implications of marginal effects for dummy variables and continuous variables are different [20]. As a continuous variable has changed, the crash frequency and severity are adversely affected, such that the elasticity indicates the effect of the corresponding factor on crash frequency at each severity level. For a dummy variable, the elasticity effects are computed by changing the value of the variable from 0 to 1 or from 1 to 0. In other words, for the subsample of observed roadway entities that the variable takes a value of 0, changing the value of variable to 1; and for the subsample that the variable takes a value of 1, changing the value of variable to 0. Then the shifts of expected frequencies would be summed up in the two subsamples after reversing the sign of the subsample, and an effective percentage change in aggregate frequency would be estimated and computed. Hence, the elasticity effects of dummy variables could be interpreted as the percentage change at the expected crash frequency due to the change in the dummy variable from 0 to 1.

Fu and Chiou [21] proposed an integrated model under the multinomial generalized Poisson (MGP) framework to analyze traffic crashes and the elasticity effects of significant variables have been reported to better understand the impact of contributing factors. The results showed that the traffic characteristics have greater effects on crash frequency and severity compared to the geometric variables. Anastasopoulos and Mannering [22] explored the usage of random-parameters models in analyzing crash frequencies. The average marginal effects were computed and the results showed that the average marginal effects generated by the standard NB and random-parameters NB models can be quite different. Wang and Kockelman [23] proposed a heteroscedastic ordered logit model to study the effects of various vehicle, environmental, roadway, and occupant characteristics on the crash severity. The elasticties were estimated to provide some insight into the implications of the estimation results. The zero-inflated count models and nested logit models were proposed by Lee and Mannering [24] to analyze run-off-roadway traffic crashes on a 96.6-km section of highway in Washington State. The analyses identified a wide range of factors that significantly contribute to the frequency and severity of run-off-roadway traffic crashes and the marginal effects of significant factors were computed to provide a guideline on the effectiveness of potential traffic safety countermeasures. It can be concluded that, even the marginal effects can provide some insights, they only estimate an average change that caused by one variable. To obtain some comprehensive results, in terms of superior predictions of traffic crashes for roadway system, a more appropriate method is needed.

As research progressed, the limitations of the statistical model quickly became obvious and machine learning methods including Artificial Neural Network (ANN), Support Vector Machine (SVM) models, and deep learning models have been proposed as methodological alternatives because of their ability to work with massive amounts of multi-dimensional data. Compared to the models that developed for traffic crash estimations, the studies that investigated the predictive models to forecast traffic crashes are relatively limited. Over the past 20 years, only a few studies have proposed the predictive models that specifically analyze the traffic crashes.

ANN and Bayesian neural network (BNN) models have been proposed to address the traffic safety problems for many years. Although ANN and BNN models have similar multilevel network structures, they are different in forecasting the traffic crashes. The weights in ANN are assumed to fix and the weights of BNN follow a probability distribution. Hence, the predictions of BNN need to be integrated over all the probability weights. Abdelwahab and Abdel-Aty [25] employed two well-known ANN paradigms to analyze the traffic safety of toll plazas. The results showed that the Radial Basis Functions (RBF) neural network was the best model for analyzing driver injury severity. Chang [26] compared the performances of NB regression model and ANN in traffic crash predictions. The results showed that ANN is a consistent alternative method for analyzing crash frequency. Xie, Lord, and Zhang [27] evaluated the application of BNN models for predicting traffic crashes by using data collected in Texas. The results showed that back-propagation neural network (BPNN) and BNN models outperform the NB regression models in terms of traffic crash prediction. Akin and Akbas [28] proposed an ANN model to predict intersection crashes in Macomb County of the State of Michigan. The results showed that ANN model is capable of providing an accurate prediction (90.9%). Kunt, Aghayan, and Noii [29] employed a genetic algorithm (GA), pattern search, and ANN models to predict the severity of freeway traffic crashes. The results showed that the ANN provided the best predictions. Jadaan, Al-Fayyad, and Gammoh [30] developed a traffic crash prediction model using the ANN simulation with the purpose of identifying its suitability for predicting traffic crashes under Jordanian conditions. The results demonstrated that the estimated traffic crashes are close to actual traffic crashes. Zeng and Huang [31] proposed a stable and optimized ANN for crash injury severity prediction. With a convex combination algorithm and a function approximation algorithm, the proposed ANN showed superior performances. Other modified ANN models including the studies of Huang et al. [32] and Zeng et al. [33, 34] also confirmed that the modified ANN models have better performances compared to the statistical models. In summary, though ANN and BNN models show better approximation properties than traditional statistical approaches, these models often cannot be generalized to other data sets [1].

The SVM models that are based on statistical learning theory and structural risk minimization have been employed for traffic safety analyses [35, 36]. Li et al. [36] evaluated the application of SVM models for predicting traffic crashes. The results showed that the SVM models predict crash data more effective and accurate than traditional NB models. In addition, the findings indicated that the SVM models provide better (or at least comparable) performance than BPNN models and do not over-fit the data. Ren and Zhou [37] proposed a novel approach that combines particle swarm optimization (PSO) and SVM for traffic crash prediction. The results showed that the predictions of PSO-SVM are better than that from BP neural network. Yu and Abdel-Aty [38] proposed the SVM models with different kernel functions to evaluate real-time crash risk. The results showed that the SVM model with RBF kernel has better performance compared to the SVM model with linear kernel and Bayesian logistic regression model. Dong, Huang, and Zheng [11] proposed a SVM model to handle multidimensional spatial data in crash prediction. The results showed that the SVM models outperform the non-spatial models in terms of model fitting and predictive performance. In addition, the SVM models provided better goodness-of-fit compared with Bayesian spatial model with conditional autoregressive prior when utilizing the whole dataset as the samples. Chen et al. [39] employed the SVM models to investigate driver injury severity patterns in rollover crashes. The results showed that the SVM models produce reasonable predictions and the polynomial kernel outperforms the Gaussian RBF kernel. Overall, it has been found that the SVM models showed better or comparable results to the outcomes predicted by ANN/BNN and other statistical models. However, like ANN and BNN, the SVM models often cannot be generalized to other data sets and they all tend to behave as black-boxes, which cannot provide the interpretable parameters as statistical models do.

Apart from the ANN/BNN and SVM models, other machine learning methods have been introduced in traffic safety analyses. Abdel-Aty and Haleem [40] employed a multivariate adaptive regression splines (MARS) to predict vehicle angle crashes. The results showed that MARS outperformed the NB models. The proposed MARS models showed promising results after screening the covariates using random forest and the findings suggested that MARS is an efficient technique for predicting traffic crashes at unsignalized intersections. Deep learning is a recently developed branch of machine learning method and has been successfully applied in speech recognition, visual object recognition, object detection, and many other domains such as drug discovery and genomics [41, 42]. Compared to the conventional machine learning techniques that were limited in their ability to process data in their raw form, deep learning construct computational models aiming to extract inherent features in data from the lowest level to the highest level. Though the deep learning methods have shown outstanding performances in many applications [41], the applications of deep learning in the field of transportation are relatively few and only focusing on the topic of traffic flow prediction [43–45].

In this study, we proposed an innovative two-step method and a support vector regression (SVR) model is formulated into SSM framework for traffic crash prediction. To identify the dynamic evolution process of the roadway systems that are caused by the changes of traffic flow, a state-space model was developed in the first step to predict the changes of impact factors in roadway systems. Using the predicted impact factors, the SVR model was incorporated in the second step to predict the traffic crashes. To validate the model effectiveness, a five-year dataset that obtained from 1152 roadway segments in Tennessee was employed and a SVR model and a multivariate negative binomial (MVNB) model were developed as the benchmark methods.

## 2. Methodologies

**Fig 1:**
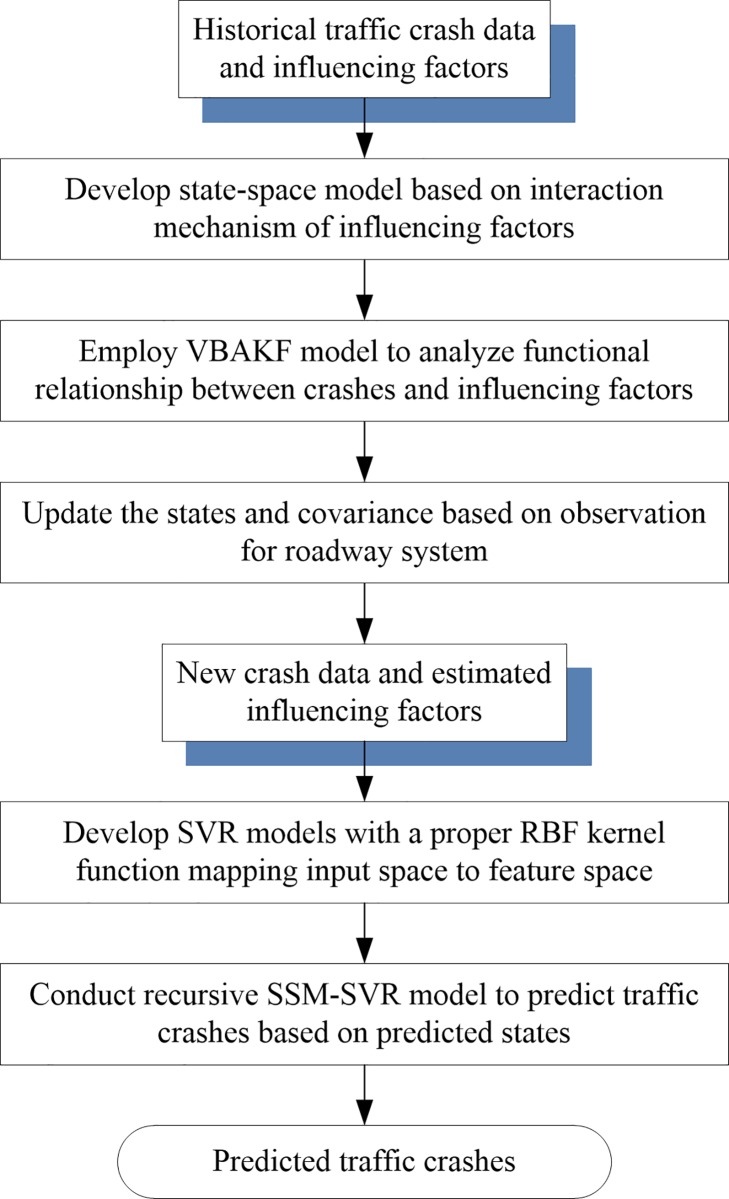
Flowchart of the proposed approach for traffic crash prediction.

Conventional traffic crash predictions ignore the changes of roadway systems, which can lead to inaccurate predictions. To address this issue, we designed a two-step method and a SVR model is formulated into SSM framework (referred to as SSM-SVR approach) for traffic crash prediction. Specifically, the state-space model is proposed to capture the dynamic evolvement between the impact variables and the control input variables for a roadway segment. The estimation results are incorporated into the SVM model to improve the prediction accuracy. The flowchart of the proposed SSM-SVR approach for traffic crash prediction is shown in Fig 1. A roadway segment is assumed to a system and the effects of impact factors on traffic crashes were captured by the state variables. With the traffic flow as the control input, the system state is changing by time. Other unobserved factors were considered as process and sensor noise in the proposed state-space model. The proposed state-space model for traffic crash prediction assumes that the state of roadway entity at time *t* evolves from the prior state at time *t*-1 according to Eq (1).

$$x_{t+1}=A_{t}x_{t}+B_{t}q_{t}+w_{t}$$ (1)

Where *x*_*t*_ is the state vector at time *t*, *q*_*t*_ is the vector containing any control inputs, *A*_*t*_ is the state transition matrix, *B*_*t*_ is the control input matrix that applies the effect of each control input parameter in the vector *q*_*t*_ on the state vector, and vector *w*_*t*_ denotes process noise that is zero-mean, white Gaussian, stochastic process with covariance matrixes **Q**.

Eq (1) reflects the dynamic evolvement between the state variables and the control input variables for a roadway segment. The roadway geometric design features, traffic control methods, pavement conditions, and environmental characteristics have been considered as the state variables. With the state transition matrix, the upgrade information can be incorporated and the current state can evolve into the next state. On the other hand, the control input variables represent the needs of upgrading.

To evaluate how the changes of geometric design features, pavement conditions, traffic control methods, and environmental characteristics affect traffic safety, the regression models have been embedded in the proposed state-space model. Assuming that *y*_*t*_ = ln*λ*_*t*_, and *λ* is the number of observed traffic crashes, the measurement equation of the roadway segment can be written as:

$$y_{t}=C_{t}x_{t}+v_{t}$$ (2)

where *C*_*t*_ is the transformation vector that maps the state vector into the measurement domain, and *v*_*t*_ is the vector that contains the measurement noise terms for each observation in the measurement vector, which is assumed to be zero mean Gaussian white noise with covariance **R**.

For *k* types of crashes, the transformation vector can be written as:

$$C_{t}=\left[\sum_{i=1}^{k}\beta_{i1},\sum_{i=1}^{k}\beta_{i2},\cdots ,\sum_{i=1}^{k}\beta_{ij}\right]$$ (3)

where *β* is an estimable parameter and *j* is the number of variables in the state vector.

The Kalman Filter (KF) [46, 47] can provide an algorithm to determine an estimate of *x*_*t*_. However, with the unknown measurement noise covariance **R**, the KF method is inappropriate. To obtain better estimates, the Variational Bayesian Adaptive Kalman Filter (VB-AKF) [48–50] is employed and the Markovian dynamic model prior distribution for the unknown measurement noise covariance can be jointly represented by the filtering distribution of the state and covariance matrix and approximated with the free-form variational Bayesian approximation as follows:

$$p(x_{t},R_{t}|y_{1:t-1})\approx Ν(x_{t}|{\hat{X}}_{t},P_{t})\mathrm{IW}(R_{t}|v_{t},V_{t})$$ (4)

where ${\hat{X}}_{t}$ and **P**_*t*_ are given by the standard KF, and *v*_*t*_ and **V**_*t*_ are the parameters of the inverse Wishart (IW) distribution.

The proposed VB-AKF algorithm is different compared to the KF algorithm. For the prediction, Eq (5) is computed after projecting the error covariance $P_{t}^{-}$. For the update process, set $v_{t}=v_{t}^{-}+1$ and the update of the measurement noise covariance, as shown in Eq (5), is added at the end.

$$R_{t}^{j+1}=(\frac{v_{t-1}-d-1}{v_{t}-d-1})R_{t}^{-}+(\frac{1}{v_{t}-d-1})H_{t}P_{t}^{j+1}H_{t}^{T}+(\frac{1}{v_{t}-d-1})(y_{t}-H_{t}{\hat{X}}_{t}^{j+1})(y_{t}-H_{t}{\hat{X}}_{t}^{j+1}{)}^{T}$$ (5)

where $v_{t}^{-}$ and $R_{t}^{-}$ are prior parameters, $v_{t}^{-}=\rho (v_{t-1}-d-1)+d+1$ and $R_{t}^{-}=BR_{t-1}B^{T}$, *ρ* is a real number and 0<*ρ*≤1, which controls the forgetting of the previous estimates of the measurement covariance matrix by decreasing the degrees of freedom exponentially, and **B** is a matrix 0<|**B**|≤1, which can be used to model the deterministic dynamics of the covariance matrix.

The proposed models estimate a state by using a form of feedback control. In other words, the filter estimates the state at some time and then obtains feedback in the form of measurements. As such, the predictor equations are projecting forward the current state and error covariance estimates to obtain the *a priori* estimates for the next time step. The measurement update equations are using for incorporating a new observation into the *a prior* estimate to obtain an improved *a posteriori* estimate. With the estimated states, a specifically designed SVR model is proposed to predict traffic crashes. In the proposed SVR model, given a set of training samples {(*x*_1_, *y*_1_), (*x*_2_, *y*_2_), …, (*x*_*N*_, *y*_*N*_)} with *x*_*i*_∈R^*d*^ and *y*_*i*_∈R, the linear regression model can be expressed as

f(x)=ωφ(x)+b (6)

where *φ*(*x*) represents the high-dimensional feature spaces, which are nonlinearly mapped from the input space *x*, *ω* = [*ω*_1_, *ω*_2_, *…*, *ω*_*N*_]^*T*^ represents the vector or regression coefficients and *b* denotes the bias. The regression problem can be solved by the following constrained optimization problem

$$\min\frac{1}{2}{\left\|\omega \right\|}^{2}+E\sum_{i=1}^{N}L^{\varepsilon }(x_{i},y_{i},f)$$ (7)

$$\begin{array}{cc} s.t. & \left\{ \begin{array}{l} y_{i}-\omega \cdot \varphi (x_{i})-b\leq \varepsilon +\xi_{i}\\ \omega \cdot \varphi (x_{i})+b-y_{i}\leq \varepsilon +\xi_{i}^{*}\\ \xi_{i},\xi_{i}^{*}\geq 0 \end{array}\right. \end{array}$$

where *E* is a regularization parameter, *ε* is the tolerance threshold and *L*^*ε*^ is a *ε*-insensitive loss function that penalizes errors larger than ±*ε*, *ξ*_*i*_ and *ξ*_i_^*^ represent slack variables to make constraints feasible.

To handle the nonlinearity in regression problem, the training sample (*x*_*i*_, *x*_*j*_) can be substituted with a kernel function *K*(*x*_*i*_, *x*_*j*_), which linearizes the relationship between *x*_*i*_ and *y*_*i*_ [51]. By introducing the Lagrange multiplies, the nonlinear optimization problem can be formulated as

$$\max‑\frac{1}{2}\sum_{i=1}^{N}\sum_{j=1}^{N}\left(\alpha_{i}-\alpha_{i}^{*})(\alpha_{j}-\alpha_{j}^{*})K(x_{i},x_{j}\right)-\sum_{i=1}^{N}(\alpha_{i}-\alpha_{i}^{*})y_{i}-(\alpha_{i}+\alpha_{i}^{*})\varepsilon$$ (8)

where *K(x*_*i*_, *x*_*j*_) denotes kernel function. The commonly used kernel functions include the linear kernel *K*(*x*_*i*_, *x*_*j*_) = *x*_*i*_-*x*_*j*_, polynomial kernel *K*(*x*_*i*_, *x*_*j*_) = (*x*_*i*_·*x*_*j*_+1)^*d*^, and the radial-basis function (RBF) kernel *K*(*x*_*i*_, *x*_*j*_) = exp(-*γ||x*_*i*_-*x*_*j*_||^2^), where *d* and *γ* are the kernel parameters. The Lagrange multiplies *α*_*i*_, *α*_i_* can be determined by solving Eq (8) with constraints and the regression function is given by

$$f(x)=\sum_{i=1}^{N}(\alpha_{i}-\alpha_{i}^{*})K(x,x_{i})+b$$ (9)

**Fig 2:**
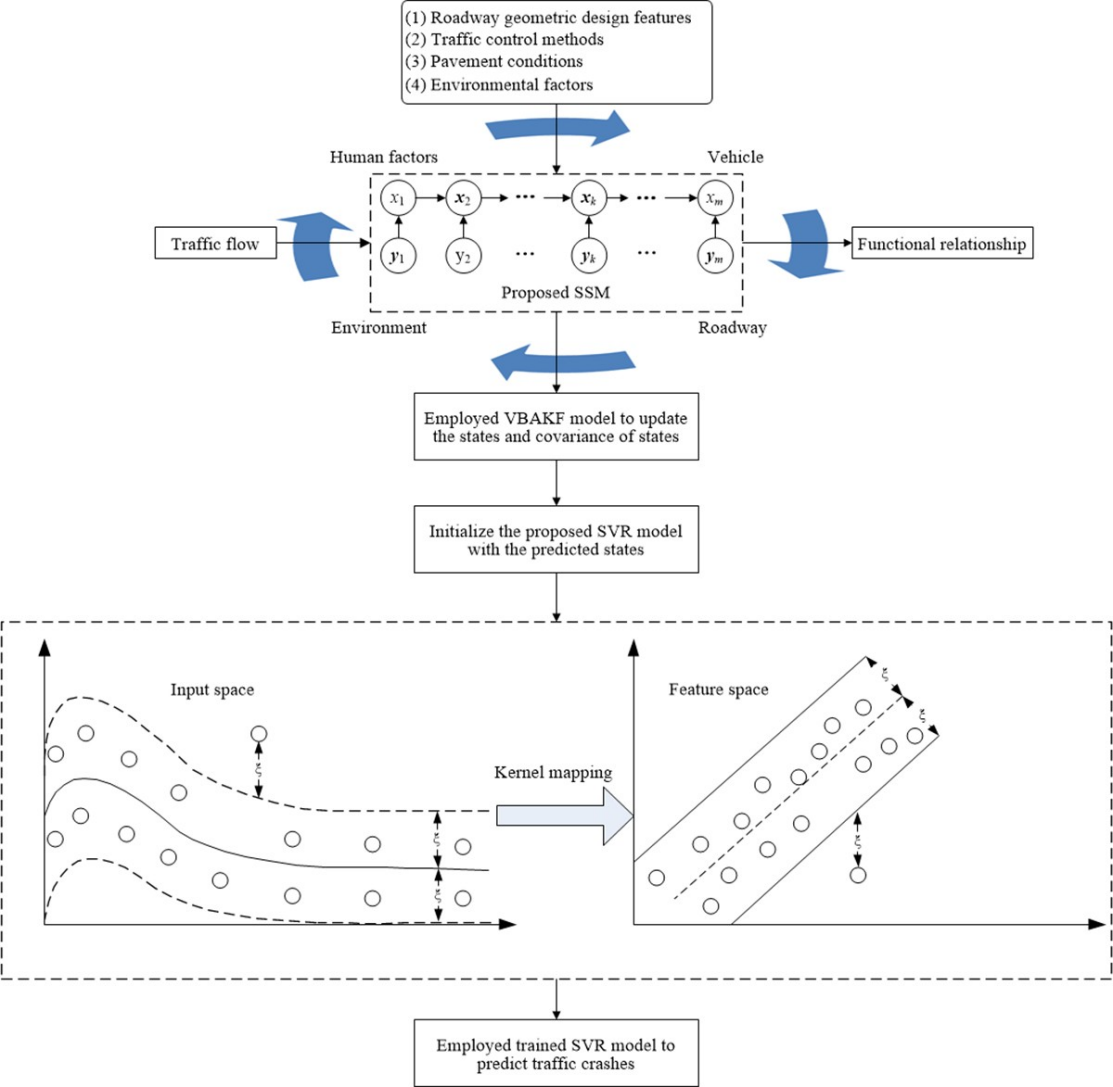
Graphical illustration of the proposed SSM-SVR approach for traffic crash prediction.

By incorporating kernel function *K*, the input *x* can be replacing by a mapping into feature space *ψ*(*x*), as shown in Fig 2. Through solving a quadratic programming problem, the proposed SVR model searches for the nonlinear regression function that is linear in high-dimensional feature space. With nonlinear kernel functions and a number of identified support vectors, the proposed SVM model is obtained to predict the traffic crashes. Because the formulation embodies the structural risk minimization principle, which has superior performances compared to the conventional empirical risk minimization principle that used in the ANN method, the proposed SVR has enhanced the ability of prediction and can avoid over-fitting issue. Another merit of the proposed SVR is that instead of minimizing the observed training error, the SVR model attempts to minimize the generalized error bound so as to achieve generalized performance.

## 3. Data description

Data that obtained from the Tennessee Department of Transportation (TDOT) are employed to assess the performances of the proposed SSM-SVR models in terms of the ability to predict traffic crashes across injury severities. We complied with the terms of service of the TDOT about the data. Two data sets, including Tennessee Roadway Information Management System (TRIMS) and Pavement Management System (PMS) are incorporated through common variable *id_number*, which is a combination of county, county sequence, route type, and route number. After screening the combined data set, state routes 001, 033, 062, 071, 115, 168, 169, and 331 that contain 1152 segments, with an average segment length of 0.82 miles were chosen for analyses. Extensive data cleaning and consistency checks were conducted and the crash data on those state route roadways from 2010 to 2014 were employed in the study. In TRIMS, the crash data have five categories, including fatal crashes, incapacitating injury crashes, non-incapacitating injury crashes, possible-injury crashes, and property damage only (PDO) crashes. Because there are significant differences in the number of crash counts across injury severities and the number of fatal crashes is much less compared to the number of other crash categories, a classification method has been used for the incorporation of injury outcomes. According to pervious literature [52–54], the fatal crashes and incapacitating injury crashes have been incorporated and referred to as major injury crashes. Correspondingly, the possible-injury crashes and PDO crashes have been incorporated and referred to as no-injury crashes. The non-incapacitating injury crashes are referred to as minor injury crashes. The final dataset includes 320 (2.74%) major injury crashes, 2879 (24.66%) minor injury crashes, and 8474 (72.59%) no-injury crashes. Individual roadway segment experienced from 0 to 29 crashes per year with a mean of 2.03 and a standard deviation of 1.79.

The road inventory records on those state route roadways including traffic factors, geometric design features, and environmental characteristics were linked to the crash data. Important measurements of traffic factors considered in this study include thousand passenger car annual average daily traffic (AADT) per lane, thousand truck AADT per lane, and posted speed limit. The thousand passenger car AADT per lane from 2010 to 2014 varies from 0.38 to 35.24 with a mean of 3.25 and a standard deviation of 1.36 and the thousand truck AADT per lane from 2010 to 2014 varies from 0.03 to 6.09 with a mean of 0.25 and a standard deviation of 0.13. The variable of posted speed limit has been considered as a categorical variable and two categories have been considered with a threshold value of 55 mph. For 49.83 percent (2870) roadway segments, the posted speed limit is less than 55 mph and 50.17 percent (2890) roadway segments, the posted speed limit is greater than 55 mph.

Eight geometric design features have been considered for analyses, since they might have potential impacts on traffic crashes. Among them, the segment length, degree of horizontal curvature, median widths, and outside shoulder width have been considered as the continuous variables and the number of through lanes, lane widths, median type, and shoulder type have been considered as the categorical variables. The variable of segment lengths varies from 0.09 to 12.57 with a mean of 0.82 and a standard deviation of 1.30. The analyzed degree of horizontal curvature varies from 0.00 to 14.42. For the variables of median widths and outside shoulder widths, the values vary from 0.00 to 11.92 and from 1.68 to 10.12, respectively. The analyzed number of through lanes have three categories, including six lanes (1465, 25.43%), four lanes (2730, 47.40%), and two lanes (1565, 27.17%). Three types of lane widths have been examined, including 12 ft. (1170, 20.31%), 11 ft. (3340, 57.99%), and 10 ft. (1250, 21.70%). The analyzed median types have been classified into three categories, including two-way left turn lanes (TELTL) (785, 13.63%), raised median (1375, 23.87%), and no median (3600, 62.50%). Three types of shoulders have been examined, including pavement (1995, 34.64%), gravel (2510, 43.58%), and dirt (1255, 21.79%).

Other than the traffic factors and geometric design features, the characteristics of pavement and environment might affect the likelihood of crash occurrences. Two pavement condition indicators, including international roughness index (IRI) and rut depth (RD) have been analyzed as continuous variables. The analyzed IRI that is calculated using a quarter-car vehicle math model varies from 25.35 to 195.90 with a mean of 76.88 and a standard deviation of 34.11. The variable of RD that is measured with a laser/inertial profilograph varies from 0.05 to 0.66 with a mean of 0.15 and a standard deviation of 0.07.

Important measurements of environmental characteristics considered in this study include terrain type, land use type, and indicator for lighting. All the environmental variables have been analyzed as categorical variables. The terrain type has been classified into two categories, mountainous terrain type (2410, 41.84%) and rolling terrain type (3350, 58.16%). Three land use types are examined, which include residential land use (2770, 48.09%), commercial land use (1695, 29.43%), and rural land use (1295, 22.48%). Two lighting conditions that indicate whether lighting devises are provided at the roadway segments were considered. The data show that 54.86 percent (3160) roadway segments have lighting devises, and the others (2600, 45.14%) don’t have lighting devices. The descriptive statistics of continuous variables and categorical variables are shown in Tables 1 and 2, respectively. We complied with the terms of service of the TDOT about the data.

**Table 1:** Summary statistics of analyzed continuous variables.

Variable	Mean	Std. Dev.	Min.	Max.
***Independent variable***				
The number of major injury crashes per year per roadway segment	0.06	0.28	0	3
The number of minor injury crashes per year per roadway segment	0.50	1.19	0	12
The number of no injury crashes per year per roadway segment	1.47	2.98	0	24
***Traffic factors***				
Thousand passenger car AADT per lane	3.25	1.36	0.38	35.24
Thousand truck AADT per lane	0.25	0.13	0.03	6.09
***Geometric design features***				
Segment length (miles)	0.82	1.30	0.09	12.57
Degree of horizontal curvature	1.67	3.55	0.00	14.42
Median widths	1.74	2.34	0.00	11.92
Outside shoulder widths	3.29	2.58	1.68	10.12
***Pavement factors***				
International roughness index	76.88	34.11	25.35	195.90
Rut depth (in.)	0.15	0.07	0.05	0.66

**Table 2:** Summary statistics of analyzed categorical variables.

Variable	Category	Frequency	Percent
***Traffic factor***			
Posted speed limits	<55 mph	2870	49.83
	≥55 mph	2890	50.17
***Geometric design features***			
Number of through lanes	6	1465	25.43
	4	2730	47.40
	2	1565	27.17
Lane widths (ft)	12	1170	20.31
	11	3340	57.99
	10	1250	21.70
Median type	Two-way left turn lanes (TWLTL)	785	13.63
	Raised median	1375	23.87
	No medians	3600	62.50
Shoulder type	Pavement	1995	34.64
	Gravel	2510	43.58
	Dirt	1255	21.79
***Environmental factors***			
Terrain type	Mountainous	2410	41.84
	Rolling	3350	58.16
Land use type	Residential	2770	48.09
	Commercial	1695	29.43
	Rural	1295	22.48
Indicator for lighting	Lighting exists on the roadway segments	3160	54.86
	No lighting devices	2600	45.14

## 4. Modelling results

**Fig 3:**
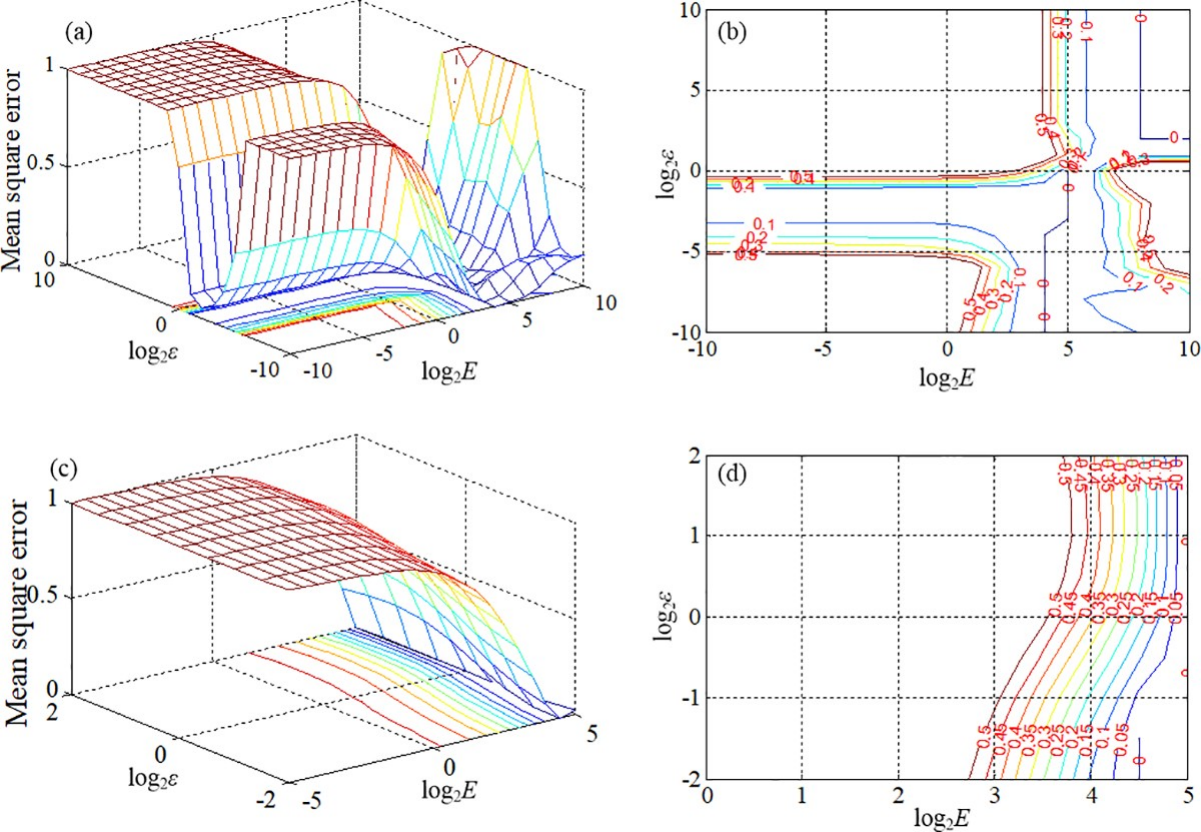
Searching process and results based on the 4-fold cross validation: (a) first search; (b) contour plot corresponding to first search; (c) second search; (d) contour plot corresponding to second search.

The data from 2010 to 2013 were used to train the proposed SSM-SVR model and the data of 2014 was used as a verifying set. The obtained coefficients from a regression model are used as the initial values for the matrixes of *A*, *B*, and *C* in the proposed models and the MATLAB was employed for model development. Kernel selection and parameters optimization are two key issues of the SVR models. The RBF kernel is chosen, since the RBF kernel can handle the non-linear relationship between impact factors and outputs with less parameter [55]. Based on the definition of -*γ* = 1/*k*, where *k* means the number of impact factors, -*γ* is correspondingly set to 1/21 in the further modeling process. To optimize the parameters of the SVR model (*E* and *ε*) and address the potential over-fitting issue, the cross-validation approach [56, 57] is employed to perform the parameter search. To identify better *E* and *ε*, the training set is divided into four subsets with equal size and one subset is tested using the predictor trained on the remaining three subsets. A grid search was performed over a pre-defined parameter space and the model with the highest prediction accuracy (i.e. lowest cross-validation error) was employed. First parameter search space have a greater range and defined from 2^−10^ to 2^10^ by experiment. The result shows the optimum parameter space is from 2^−5^ to 2^5^ for *E* and from 2^−2^ to 2^2^ for *ε* with the converged mean square error (MAE) of 0.39. Second parameter search space is performed based on the results of first search. With a MAE of 0.23, the results show that final optimized *E* converging to a value of 22.63 and *ε* converging to a value of 0.25. The searching process and results for the proposed model are shown in Fig 3. The optimized SVR model was used as part of the proposed SSM-SVR model to perform the traffic crash prediction.

To verify the effectiveness of the proposed SSM-SVR model, other than comparing to the observed values, a SVR model and a MVNB model were developed as the benchmark methods. The RBF kernel is also adopted by the developed SVR model and the same searching process is used for parameter optimization. Final model yields to a prediction MAE of 0.22 with a *E* of 34.56 and a *ε* of 3.55. The developed SVM and MVNB models have been used to predict the traffic crashes for the analyzed 1152 roadway segments. Three commonly used indexes, including Mean Absolute Percentage Error (MAPE), Mean Absolute Error (MAE), and Root Mean Squared Error (RMSE) have been used to assess the model performances in terms of prediction accuracy and robustness.

To predict the traffic crashes in 2014, we are assuming that the values of impact factors in 2014, including geometric design features, traffic control methods, pavement conditions, and environmental characteristics are unknown. The traffic flow variables including thousand passenger car AADT per lane and thousand truck AADT per lane can be estimated. For the proposed SSM-SVR model, the prediction part used the estimated traffic flow variables and predicted state variables that obtained from the SSM as the input variables. For the developed SVR and MVNB models, the values of impact factors in 2013 and estimated traffic flow variables were used as the input variables. Table 3 shows the comparison between the observation in 2014 and the inputs for the developed models. Compared to the current observation in 2013 (input for the SVR and MVNB models), the predicted state variables from SSM (input for the proposed SSM-SVR model) are closer to the observation in 2014. In other words, using predicted impact factors is a better alternative for the prediction when the further roadway conditions are unknown. Using current observation in 2013 and predicted state variables that are obtained from SSM to substitute the real values of observation in 2014 produces an average MAPE of 9.17% and 4.67%, respectively.

**Table 3:** Comparison between the observation in 2014 and the inputs for the developed models.

Variable	Observed value in 2014	Input for SVR and MVNB models	MAPE (%)	Input for the proposed models	MAPE (%)
***Continuous variables***					
Thousand passenger car AADT per lane	3.67	3.30	10.08	3.83	4.33
Thousand truck AADT per lane	0.28	0.27	5.36	0.29	2.86
Segment length (miles)	0.82	0.82	0.11	***0*.*82***	0.54
Degree of horizontal curvature	1.67	1.67	0.05	1.63	2.58
Median widths	1.66	1.87	12.79	1.70	2.36
Outside shoulder widths	3.00	3.14	4.89	3.05	1.78
International roughness index	69.87	73.38	5.02	68.91	1.37
Rut depth (in.)	0.14	0.15	4.20	0.15	3.96
***Categorical variables***					
Posted speed limits <55 mph	534	466	12.73	573	7.30
≥55 mph	618	686	11.00	579	6.31
Number of through lanes = 6	325	381	17.23	355	9.23
= 4	511	490	4.11	470	8.02
= 2	316	281	11.08	327	3.48
Lane widths (ft) = 12	210	191	9.05	202	3.81
= 11	668	641	4.04	677	1.35
= 10	274	320	16.79	***273***	0.36
Median type = two-way left turn lanes (TWLTL)	157	169	7.64	165	5.10
= raised median	249	206	17.27	260	4.42
= no medians	746	777	4.16	727	2.55
Shoulder type = pavement	333	281	15.62	326	2.10
= gravel	442	432	2.26	406	8.14
= dirt	377	439	16.45	420	11.41
Terrain type = mountainous	528	452	14.39	495	6.25
= rolling	624	700	12.18	657	5.29
Land use type = residential	630	647	2.70	624	0.95
= commercial	273	235	13.92	300	9.89
= rural	249	270	8.43	228	8.43
Indicator for lighting = lighting exists on the roadway segments	638	702	10.03	606	5.02
= no lighting devices	514	450	12.45	546	6.23

The prediction results are shown in Table 4.The results show that the proposed SSM-SVR models have superior performances compared to the SVR model and MVNB model. The predicted distribution of the proposed SSM-SVR model is closed to the observed distributions that measured by the summary statistics of distribution. The predicted means of the proposed SSM-SVR model are 0.043, 0.417, and 0.919 for major, minor, and no-injury crashes, respectively, which are closed to the observed means of 0.049, 0.385, and 0.997. In addition, the predicted distribution ranges that are measured by minimum values and maximum values are as same as the observed values for all crash types. The SVR model and MVNB model have comparable performances. The predicted means of the SVR model are 0.056, 0.432, and 1.110 and the predicted mean of the MVNB model are 0.057, 0.440, and 1.135. Though the SVR model performs better compared to the MVNB model in terms of prediction accuracy, the MVNB models have better robustness. The predicted maximum values from MVNB model are 5, 10, and 21 for major, minor, and no-injury crashes, respectively, which are closed to the observed values compared to the predicted values from SVR model.

**Table 4:** Results of traffic crash prediction.

	Major injury crashes	Minor injury crashes	No-injury crashes	Total
**Observation**				
Observed mean	0.049	0.385	0.997	1.430
Observed Std. Dev.	0.302	1.102	2.095	2.350
Observed min	0	0	0	0
Observed max	3	10	19	19
Observed counts	56	443	1148	1647
**SSM-SVR approach**				
Predicted mean	0.043	0.417	0.919	1.379
Predicted Std. Dev.	0.295	1.115	2.092	2.343
Predicted min	0	0	0	0
Predicted max	3	10	19	19
Predicted counts	50	480	1059	1589
MAPE (%)	10.714	8.352	7.753	3.522
MAE	0.005	0.060	0.173	0.215
RMSD	0.072	0.245	0.467	0.512
**SVR model**				
Predicted mean	0.056	0.432	1.110	1.599
Predicted Std. Dev.	0.314	1.134	2.222	2.472
Predicted min	0	0	0	0
Predicted max	3	9	21	22
Predicted counts	65	498	1279	1842
MAPE (%)	16.071	12.415	11.411	11.840
MAE	0.018	0.195	0.496	0.593
RMSD	0.135	0.442	0.728	0.851
**MVNB model**				
Predicted mean	0.057	0.440	1.135	1.632
Predicted Std. Dev.	0.404	1.108	2.167	2.445
Predicted min	0	0	0	0
Predicted max	5	10	21	21
Predicted counts	66	507	1307	1880
MAPE (%)	17.857	14.447	13.850	14.147
MAE	0.021	0.398	0.548	0.780
RMSD	0.177	0.700	0.771	1.123

The accuracy loss of the MVNB models can be explained by the unpredictable part of their standard specification forms, which cannot adequately address stochastic changes in the evolution process of roadway systems. Compared to the MVNB models, the SVR models can better handle the stochasticity and nonlinearity of the spatial-temporal evolution of the traffic crashes. The results can be explained by the adaptive tuning of the parameters and the dynamic mapping between the input and output. However, the SVR models do have the limitations, such as large variances in the prediction results, which demonstrate the lack of consistency and robustness. A possible explanation is that the SVR models can implicitly embed time correlation in the hidden layer. When the model is trained with two crash data with different patterns, the results can be an intermediate estimation that leads to inaccurate predictions for all crash data. After further examination, the SVM models are often found to highly overpredict or underpredict traffic crashes when crash counts have large variations. Overall, the performances of MVNB and SVR models are not robust when the data meet certain conditions, such as extra zero counts that are commonly found in the traffic crash data. The problem is solved with the proposed SSM-SVR models, which can capture almost all observed values in the validation data set.

The findings show that the predictions from the proposed SSM-SVR models have significant improvements over the SVR and MVNB models, in both accuracy and robustness. The predicted crash counts of the proposed SSM-SVR model are 50, 480, and 1059 for major, minor, and no-injury crashes, which mean a prediction MAPE of 10.714%, 8.352%, and 7.753%. The overall performances of the proposed SSM-SVR models for all crashes show an extra 8.318% improvement over the SVR models and an 10.625% improvement over the MVNB models. It is clear that the predictions obtained from the proposed SSM-SVR models are superior to those that are obtained from the SVR and MVNB models. The greatest difference is demonstrated for the major injury crashes where the proposed SSM-SVR model yields a MAPE of 10.741% compared to a MAPE value of 16.071% from the SVR models and a MAPE value of 17.857% from the MVNB models. The differences in the models for the minor injury crashes and no-injury crashes are also very important. The differences between the proposed SSM-SVR model and the MVNB model are 6.095% and 6.098% for minor injury crashes and no-injury crashes.

The proposed SSM-SVR models have better performances in terms of small error variances than the comparison models, since the proposed approach incorporates a state-space model to estimate the changes of roadway systems and a SVR model to identify the relationship between the impact factors and traffic crashes. The best-performing result of the proposed SSM-SVR model for major injury crashes yields a MAE of 0.005 and a RMSD of 0.072. The proposed SSM-SVR model performs worse for no-injury crashes, with a MAE of 0.173 and a RMSD of 0.467. However, it is still better than the predictions resulting from the SVR model and MVNB model. Compared to the SVR model, the MAE improvement of the proposed SSM-SVR model ranges from 1.869 to 2.500 with a mean of 2.210, and the RMSD improvement ranges from 0.063 to 0.261 with a mean of 0.174. Compared to the MVNB model, the MAE improvement of the proposed SSM-SVR model ranges from 2.171 to 5.638 with a mean of 3.603, and the RMSD improvement ranges from 0.652 to 1.859 with a mean of 1.320. Clearly, the improvement is significant for the proposed SSM-SVR models over the SVR and MVNB models. Therefore, the proposed SSM-SVR model seems to be a superior alternative for traffic crash predictions.

**Fig 4:**
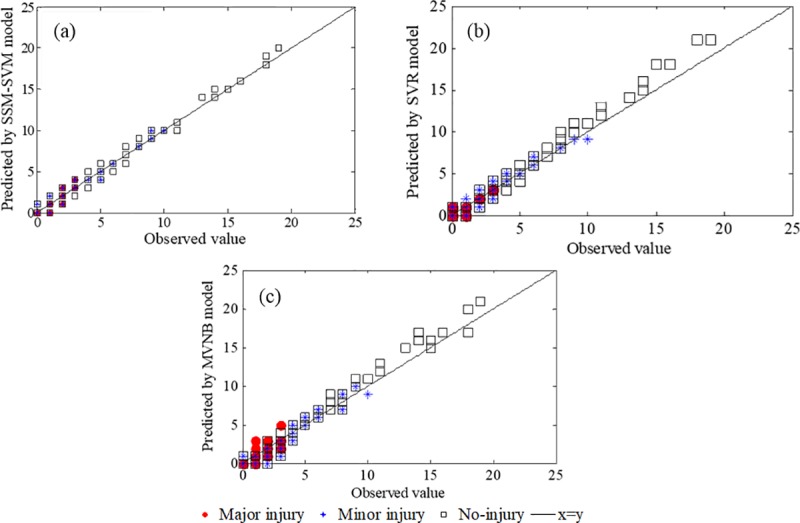
Comparison of model performances. (a) Predicted value of proposed SSM-SVR model vs. observed value. (b) Predicted value of SVR model vs. observed value. (c) Predicted value of MVNB model vs. observed value.

The result demonstrates that the proposed SSM-SVR models are better than the SVR and MVNB models, which ignores the potential changes of roadway systems. Compared to the SVR and MVNB models, the benefit of incorporating a state-space model to identify the changes of roadway systems is significant evident in the proposed SSM-SVR models for all crash types, and the prediction accuracy that measured by MAPE can be improved by 4.360% and 6.445% on average, respectively. Apart from accuracy improvement, the proposed SSM-SVR models are more robust and the predictions can retain a smoother pattern. When incorporating the combination characteristics of state-space model and SVR model, the use of the predicted impact factors as the input is found to be meaningful in the prediction. There is an apparent improvement in the results when using predicted impact factors. Table 4 shows some improvements are achieved by including the predicted impact factors for the traffic crash predictions by using the SVR models, and this is also found to be the case when the predicted impact factors are incorporated into the MVNB models. In addition, compared to the MVNB model, the SVR model has better performance. The predicted MAPE, MAE, and RMSD can be improved by 2.085%, 0.086, and 0.114 on average. The predicted results are compared to the observed values and the results are shown in Fig 4.

As it is shown in Fig 4, the proposed SSM-SVR model can capture the general trend of the traffic crashes and timely reflect a portion of the upward and downward shifts in the trend. In addition, the proposed SSM-SVR model enables the predicted traffic crashes to retain a smoother pattern, which is less sensitive to the high-frequency variations of traffic crashes, in contrast to the pattern obtained from the SVR and MVNB models. In other words, we believe that the robustness of the proposed SSM-SVR models is an advantage. The predictions obtained by the proposed SSM-SVR model are more responsive to the actual variations of traffic crashes and, hence, more accurate in comparison to the predictions obtained by the SVR and MVNB models. This is because the proposed SSM-SVR model incorporates information in both the sate-space model and SVR model and identifies dependence effects of the roadway systems. The accuracy gain, resulting from the proposed SSM-SVR models, increases in those cases where the performances of the state-space model are also increased. The prediction results show that proposed SSM-SVR model has a more precise and synchronized response behavior to the high variations of the observed data, especially for the phenomenon of extra zeros. The performances of individual predictors show that the SVR and MVNB models provide a responsive, but time-delayed representation of the fluctuation pattern, resulting in higher prediction errors compared to the proposed SSM-SVR models.

## 5. Conclusions

To perform a comprehensive analysis that aims to predict traffic crashes and alternatively, reduce traffic crashes and enhance traffic safety and operation efficiencies, an innovative two-step method that incorporates a SVR model into a SSM framework is proposed for traffic crash prediction. To describe the dynamic evolution process of the roadway systems, a state-space model was developed in the first step. With the traffic flow as the control input, the system state is changing by time and the effects of impact factors on roadway system were captured by the state variables. The SVR model was formulated into the second step to predict the traffic crashes by using the predicted impact factors from the state-space model. The performances of the proposed models were verified by using a five-year dataset that obtained from 1152 roadway segments in Tennessee and comparing to a SVR model and a MVNB model. The results and findings provide sufficient evidence for the following conclusions:

1. The results show that the proposed model has superior performances in terms of prediction accuracy compared to the SVR and MVNB models. The proposed models result in an average prediction MAPE of 7.59%, a MAE of 0.11, and a RMSD of 0.32. The overall performances of the proposed SSM-SVR models for all crashes show an extra 8.318% improvement over the SVR models and an 10.625% improvement over the MVNB models that measured by MAPE.
2. Compared to the SVR and MVNB models, the benefit of incorporating a state-space model to identify the changes of roadway systems is significant evident in the proposed SSM-SVR models for all crash types, and the prediction accuracy that measured by MAPE can be improved by 4.360% and 6.445% on average, respectively.
3. The proposed SSM-SVR models are more robust and the predictions can retain a smoother pattern. The results show that the proposed SSM-SVR model has a more precise and synchronized response behavior to the high variations of the observed data, especially for the phenomenon of extra zeros.

The findings suggest that the proposed model is a superior alternative for traffic crash predictions. Other machine learning methods can be incorporated into the proposed framework for traffic crash prediction. However, the characteristics of traffic crashes, such as the integer nature with a significant amount of zeros, might be problematic for the application of other machine learning methods. Further investigation of the proposed models includes the improvement of prediction accuracy with other supplemental machine learning methods.
