# Correction: Roadway traffic crash prediction using a state-space model based support vector regression approach

**DOI:** 10.1371/journal.pone.0223223

**Published:** 2019-09-24

**Authors:** The PLOS ONE Staff

The are errors in the Funding statement. The correct Funding statement is as follows: The funding was provided by the Fundamental Research Funds for the Central Universities (Grant No. 2019RC027). The publisher apologizes for the errors.
